# Editorial: Training in sports: the role of artificial intelligence and machine learning

**DOI:** 10.3389/fspor.2025.1590162

**Published:** 2025-04-23

**Authors:** Carlos Eduardo Gonçalves, Ernesto Costa

**Affiliations:** University of Coimbra, Coimbra, Portugal

**Keywords:** sport, machine learning, strengths and constraints, algorithms, opportunities

**Editorial on the Research Topic**
Training in sports: the role of artificial intelligence and machine learning

Artificial Intelligence (AI) and Machine Learning (ML) have permeated nearly all sectors of society, and Sport Sciences are no exception. Public discourse about AI tends to be polarized, portraying it either as a significant advancement that boosts human productivity or as a threat to job security, creativity, and human agency. Given the extensive media coverage surrounding sports, this dichotomous narrative can obscure the true implications and nuanced potentials of AI applications, which are still in their early stages in the domains of sports training and management.

The potential applications of AI in sports are extensive and appear almost boundless, encompassing diverse fields such as training load assessment and planning, injury prevention, performance analytics, player recruitment, tactical strategy development, decision-making enhancement, video analysis, and biomechanics, among others.

However, as AI algorithms become increasingly complex and opaque, a new division of labor emerges between those who develop and train these algorithms and those who utilize pre-developed solutions. Sports scientists and coaches are generally not experts in machine learning, and consequently, they might find themselves faced with AI tools that do not adequately meet their specific requirements.

Given this context, the need for thoughtful and critical examination becomes both necessary and urgent. With these considerations in mind, we present this special issue on “AI and Sport Training”. Our goal is to foster a community of researchers from both Sport and Computer Sciences, encouraging critical dialogue, theoretical exploration, and practical advancement in the intersection of these disciplines.

This special issue features four articles: two that critically address the challenges posed by AI from the perspectives of sports medicine and coaching, and two others focused on practical applications in sports physiology and video analysis.

In the first article, Naughton et al. examine the challenges and opportunities that AI presents to sports. They argue that emerging technologies will induce structural changes, enhancing productivity in multidisciplinary teams but also raising concerns about job displacement. The authors emphasize that the inevitable coexistence of humans and AI requires innovative intellectual and practical approaches.

Continuing along this reflective path, Sperlich et al. conduct a SWOT (Strengths, Weaknesses, Opportunities, and Threats) analysis regarding the application of AI in sport research, coaching, and athletic training. The authors highlight the importance of fully understanding these SWOT factors to successfully integrate AI's advantages and mitigate associated risks, thus ensuring maximal benefit from AI adoption.

On the practical application front, Heidari et al. explore how AI can optimize knowledge extraction from sports video data. Their research focuses on Zero-Shot Learning (ZSL) methods for assessing player similarity, showing promising results. They conclude that part-based models demonstrate significant advantages compared to holistic models.

Cheng et al. investigate the use of cardiorespiratory signals to estimate the Rate of Perceived Exertion (RPE) through various machine-learning models. Their findings highlight the superior accuracy of the random forest model when integrating respiratory and cardiovascular data, providing practical insights for sports physiology.

Despite the limited number of articles, this special issue represents an essential initial step toward comprehending AI's impact on sport sciences and coaching. The opportunities are significant, and dismissing AI outright due to perceived threats to human agency would be shortsighted. Equally risky is underestimating the potential negative consequences of reducing human involvement in coaching roles. As demonstrated by the contributions presented in this issue, AI tools offer powerful capabilities that sports scientists and coaches must master, ensuring their beneficial and ethical integration to enhance athletic performance and enrich the overall sporting experience.

In conclusion, AI and ML are increasingly central to sports sciences, driving innovations in athlete performance, injury prevention, and tactical analysis. While promising, the continued adoption and refinement of these technologies require overcoming challenges in data management, ethical use, and model interpretability. Within the context of this journal, we anticipate addressing these critical issues more thoroughly in a forthcoming special issue.

